# The double-edged sword of mesh use in pelvic organ prolapse surgery: a case report

**DOI:** 10.1097/MS9.0000000000001531

**Published:** 2023-12-04

**Authors:** Fawaz Khalid Alibrahim, Sarah M. AlMohaisen, Wael Sulaiman Almajed, Mohand Ali Alzughaibi, Abeer Alasiry, Mohammad Alghafees, Belal Nedal Sabbah

**Affiliations:** a Department of Urology, King Abdullah bin Abdulaziz University Hospital; b Department of Urology, King Abdulaziz Medical City; c College of Medicine, Alfaisal University, Riyadh, Saudi Arabia

**Keywords:** mesh-augmented repair, pelvic organ prolapse, pelvic organ prolapse surgery

## Abstract

**Introduction:**

Pelvic organ prolapse (POP) is a prevalent condition among parous women, often warranting surgical intervention. This case accentuates the complications associated with mesh in POP surgeries, iterating the imperative need for an evidence-based approach towards its utilization and exhaustive patient counselling.

**Case presentation:**

A 60-year-old female, post-mesh-augmented POP repair, embarked on a 13-year journey characterized by persistent pelvic pain and multiple interventions. Despite undergoing several surgeries across different countries, involving mesh and stone removals, her symptoms, notably pelvic pain and dyspareunia, persisted. Clinical examinations revealed mesh erosion into the perivesical tissue, bladder, and associated stones, which were addressed through multiple interventions, albeit with transient success.

**Discussion:**

The complex journey of this patient exemplifies the intricate challenges mesh poses in POP surgeries. While mesh application offers a minimally invasive approach and has proven successful in numerous cases, it simultaneously opens a Pandora’s box of potential severe complications, necessitating thorough patient counselling and post-surgery management.

**Conclusion:**

The case delineates the challenging path that clinicians and patients tread when navigating through mesh-associated complications post-POP repair. Although mesh has been heralded as a revolutionary approach in POP surgeries, its potential drawbacks necessitate judicious application, ensuring clinicians are well-versed with its associated risks and are adept in managing ensuing complications.

## Introduction

Highlights

- Rising concerns over mesh use in pelvic organ prolapse (POP) surgeries: Despite the promising initial adoption of non-absorbable materials like mesh in pelvic floor reconstructive surgeries, numerous complications have been reported, with mesh erosion being the most common. Regulatory bodies, including the FDA, have released advisories highlighting concerns and in some instances, restricted or prohibited the use of such meshes in specific procedures.
- Complex case of persistent complications: A 60-year-old female experienced prolonged pelvic pain and dyspareunia post-mesh use in an anterior repair surgery. Despite multiple interventions over several years, including surgeries in different countries and multiple cystoscopies, complete resolution of her symptoms remained challenging, exemplifying the intricate issues associated with mesh-augmented repairs.
- Balanced perspective on mesh use: While there’s significant discourse and warnings around the complications of mesh in POP surgeries, there’s also acknowledgement of its potential benefits. The need for thorough patient counselling, comprehensive clinician knowledge, and a balanced approach weighing both benefits and risks of mesh use in POP surgeries is emphasized.

Pelvic organ prolapse (POP) is a condition that impacts nearly half of all women who have given birth. Of these, about 11% will seek surgical intervention due to symptoms like incontinence, challenges in voiding, and the presence of a vaginal bulge^1^.

In the past 20 years, there’s been a notable surge in the adoption of non-absorbable materials in pelvic floor reconstructive surgeries^2^. Mesh emerged as a promising solution for urinary stress incontinence. Its minimally invasive nature combined with readily available kits led to a significant uptick in such procedures, undertaken by both urologists and gynaecologists. Yet, this rapid embrace of synthetic materials was not without its pitfalls, leading to a spectrum of complications^3^.

These complications, ranging from immediate post-operative issues to problems arising several years post-insertion, can be severe^4^. Recognizing the gravity of the situation, the US Food and Drug Administration (FDA) released advisories in 2008 and 2011. They highlighted concerns like mesh erosion, pain, infections, bleeding, dyspareunia, organ perforation, and urinary challenges. In 2011, the FDA even asserted that “serious complications linked to surgical mesh for transvaginal POP repair are not uncommon”^5^. Mesh erosion stands out as the most frequently reported adverse effect. Given these concerns, health authorities like the National Institute for Health and Care Excellence (NICE) in the UK and the FDA in the US, among others, have restricted or prohibited the use of PP mesh in certain prolapse procedures^6^. We present this case that exemplifies the intricate challenges and multifaceted outcomes associated with mesh use in POP surgeries. This case was reported in line with the SCARE guidelines^7^.

## Case presentation

A 60-year-old female, known case of hypothyroidism, and a history of three childbirths, visited our clinic complaining of persistent pelvic pain and dyspareunia for the past 13 years. She traced the onset of these symptoms to a cystocele she had developed, which was treated with an anterior repair using mesh in Jordan 15 years prior. Unfortunately, two years post-surgery, she experienced bladder stones and continued pelvic discomfort. In her quest for relief, she underwent medical procedures in 2015 in Jordan and 2019 in Germany. Both interventions involved cystoscopy and cystolithilolapaxy, during which portions of the stone and mesh were extracted. Yet, her pelvic pain and dyspareunia remained unresolved.

**Figure 1:**
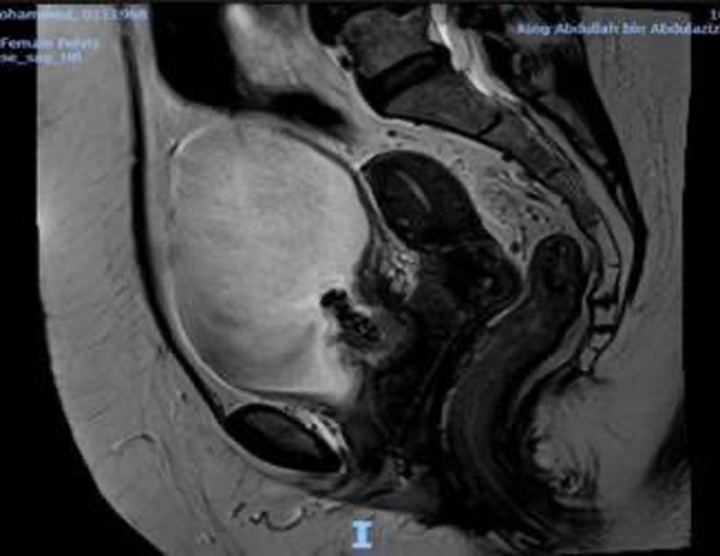
An enhanced pelvic MRI showed a defect in the base of the urinary bladder in the midline measuring ~1.2×2 cm containing heterogeneous material likely representing mesh.

Clinical examination revealed an intact anterior vaginal wall without any signs of prolapse. However, a discernible mesh and induration were present in the anterior vaginal region. No urinary leakage or urethral hypermobility was observed. Preliminary tests, including a urine culture and basic lab investigations, were conducted. The urine culture showed no significant bacterial presence, and both the electrolyte profile and renal function test results were within standard parameters. An MRI was performed to ascertain the mesh’s position, revealing a defect in the urinary bladder’s base. This defect, measuring ~1.2×2 cm, contained material likely indicative of the mesh, which seemed to have eroded into the perivesical tissue, creating a cavity of about 2.7×4.8 cm (Fig. 1).

**Figure 2:**
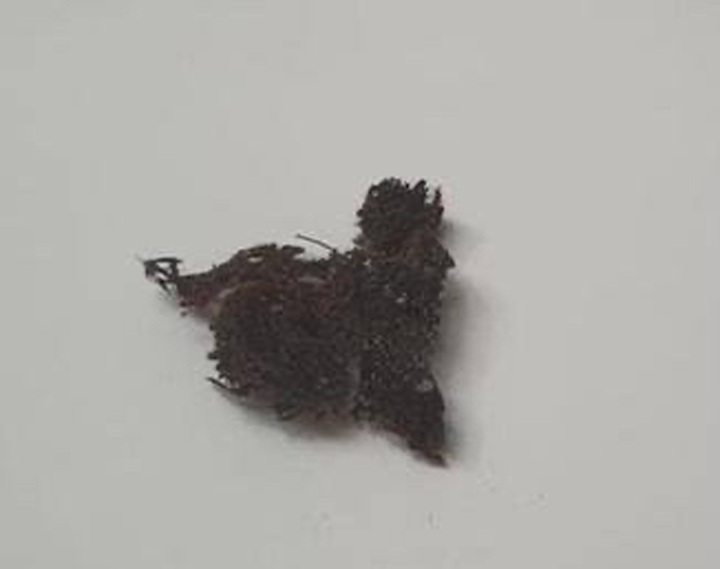
Photograph of the mesh removed after surgery.

The patient then underwent a cystoscopy, which showed mesh erosion in the triagone area accompanied by encrustation and stones. These stones were subsequently fragmented using a laser. A transvaginal incision was made, followed by the removal of small mesh fragments (Fig. 2). A subsequent cystoscopy revealed residual mesh in the bladder. A Pfannenstiel incision was then performed, leading to the bladder being bivalved, mesh excision, and the bladder and vagina being sutured in two layers each. Post-surgery, a 20 fr Foley Catheter and a JP surgical drain were placed.

**Figure 3:**
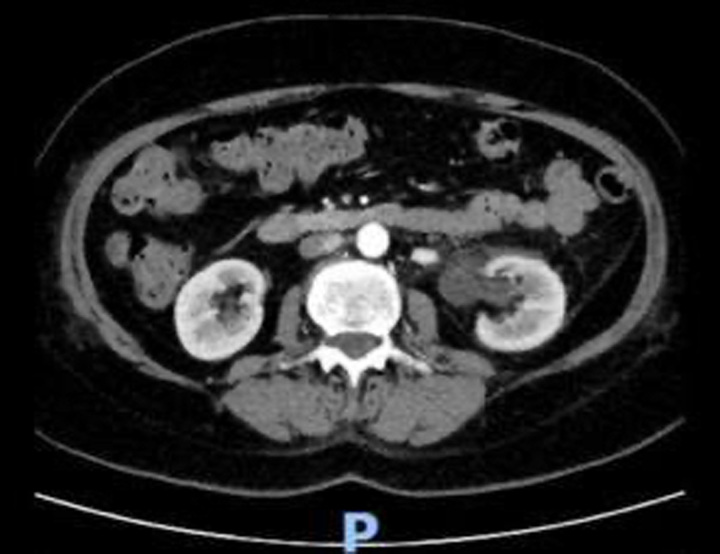
Computed tomography abdomen and pelvis pre-IV and post-IV contrast showed moderate left hydroureteronephrosis with perinephric/periuretericfat stranding and pocket of free fluid within the left anterior pararenal space.

Post-surgery, the patient’s condition was stable. However, on the third day, she experienced flank pain. Diagnostic tests, including an ultrasound and a computed tomography (CT) scan, were conducted, revealing left hydroureteronephrosis and other complications (Fig. 3). This hydroureteronephrosis likely developed secondary to oedema from the repair of the bladder wall adjacent to the left ureteral orifice. Follow-up CT scans demonstrated spontaneous resolution of the hydronephrosis. The JP surgical drain was removed, and the patient was discharged on the seventh day with the Foley catheter in place.

**Figure 4:**
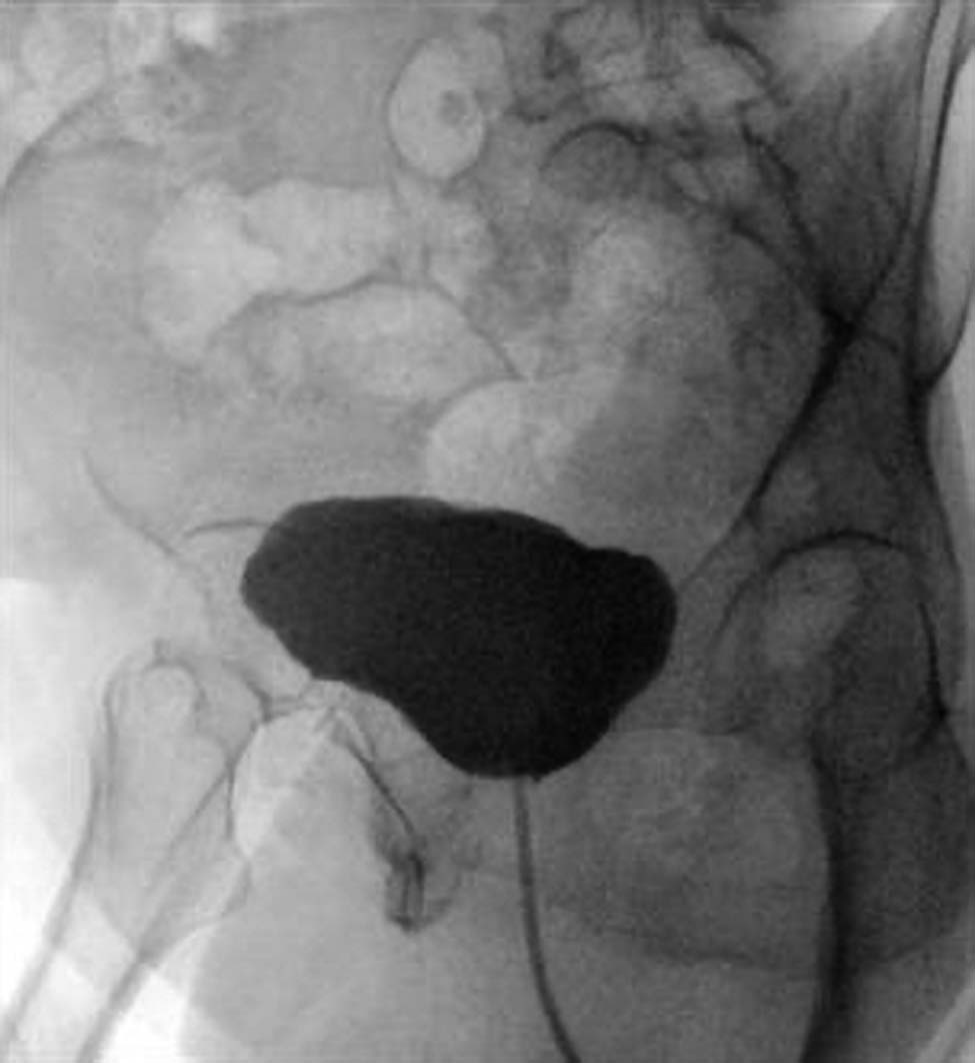
A cystography examination showed smooth well defined bladder outlines. No contrast leakage or VVF was seen VVF, Vesicovaginal Fistula.

**Figure 5:**
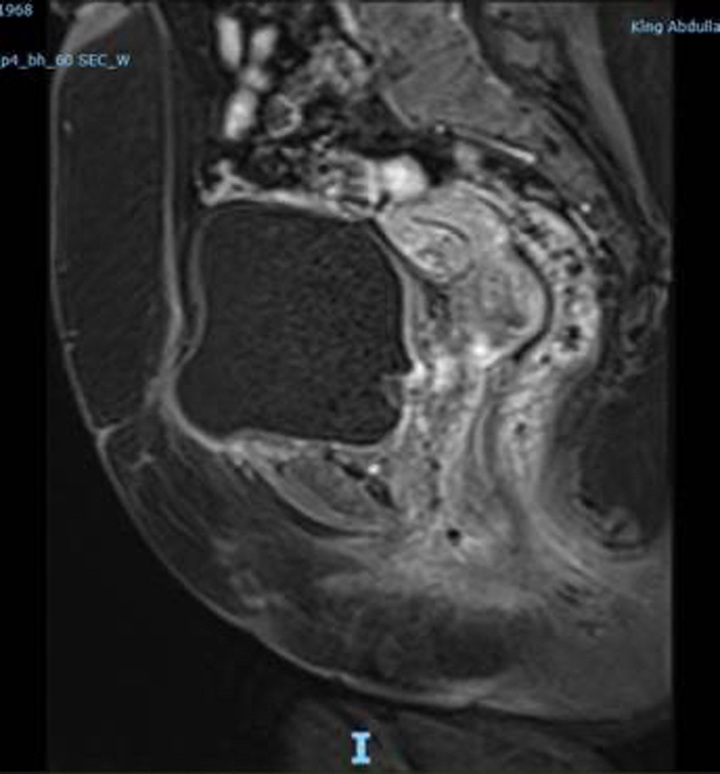
An enhanced pelvic MRI status post removal of the mesh showed small residual fibrosis and granulation tissue in that region with tethering of the posterior bladder wall. No definite leak or signs of fistula.

A month later, during her follow-up visit at the urology outpatient clinic, she reported symptoms of urgency and urge incontinence. Further investigations, including a cystoscopy and MRI, were conducted, leading to another cystoscopy procedure to address the issues (Figs. 4 and 5).

The cystoscopy showed no evidence of mesh or foreign material. A mucosal growth was identified at the site of the previously excised mesh on the right side. This growth was successfully resected using a resectoscope, and adequate haemostasis was achieved following the procedure. The patient was discharged, and the Foley catheter was removed 2 days later.

Two weeks post-discharge, she reported mild terminal dysuria but no other significant symptoms. A urine culture confirmed no bacterial infection. The patient was reassured and scheduled for a subsequent follow-up.

## Discussion

The presented case underscores the complexities and potential complications associated with the use of mesh in POP surgeries. The patient’s prolonged journey of seeking relief from persistent symptoms, despite multiple interventions, mirrors the challenges faced by many women who undergo mesh-augmented repairs.

Mesh use in POP surgeries has been a topic of significant debate in the medical community. While mesh has been employed as a promising solution for POP, its complications have led to concerns^8–10^. A study by Ridgeway *et al*.^11^ highlighted that out of patients who underwent mesh removal due to complications, most cited multiple reasons, including chronic pain, dyspareunia, recurrent POP, mesh erosion, and vesicovaginal fistula. Importantly, most of these patients reported significant relief of symptoms post-mesh removal^11^. Similarly, Blandon *et al*. emphasized that the use of vaginal mesh can lead to complications, including mesh erosions, dyspareunia, and recurrent prolapse^12^. Stanford *et al*.^13^ further noted that mesh-augmented repairs might have a higher rate of complications, especially mesh erosion/exposure, compared to traditional/native tissue repairs.

While numerous studies highlight negative outcomes, many others report significantly positive results and an enhanced quality of life^10,14^.

However, it is crucial to understand that while complications are evident, the management of mesh-related complications is equally challenging. Tijdink *et al*.^15^ emphasized that while surgical treatment of mesh-related complications is effective, it carries a substantial risk of severe complications and recurrence of POP or stress urinary incontinence. This aligns with our patient’s experience, where despite multiple interventions, complete resolution was elusive.

The FDA’s warnings on mesh-related complications have been pivotal in shaping the discourse around mesh use in POP surgeries. However, one paper suggested that the FDA’s warning might not be entirely accurate and emphasized the importance of reviewing the literature before making recommendations^16^. This highlights the need for a balanced approach, considering both the benefits and potential risks of mesh use.

## Conclusion

While mesh offers a solution for POP, its potential complications cannot be overlooked. It is imperative for clinicians to be well-informed about the risks, ensure thorough patient counselling, and be prepared for the challenges of managing mesh-related complications. The journey of our patient serves as a testament to the complexities of mesh use in POP surgeries and underscores the need for continued research and dialogue in this domain.

## Ethical approval

IRB waived.

## Consent

Written informed consent was obtained from the patient for publication of this case report and any accompanying images.

## Sources of funding

This research did not receive any funding from institutions in the public, commercial, or not-for-profit sectors.

## Author contribution

All authors contributed to the research and/or preparation of the manuscript. F.K.A., S.M.A., W.S.A., M.A.A., A.A., M.A., B.N.S. wrote the first draft of the manuscript. B.N.S. reviewed and finalized the manuscript. All authors read and approved the final manuscript.

## Conflicts of interest disclosure

The authors declare no conflict of interest.

## Research registration unique identifying number (UIN)

N/A.

## Guarantor

Mohammad Alghafees.

## Data availability statement

Confirm.

## Provenance and peer review

Not commissioned, externally peer-reviewed.
